# No Significant Effect of the Individual Chronotype on the Result of Moderate Calorie Restriction for Obesity—A Pilot Study

**DOI:** 10.3390/nu13114089

**Published:** 2021-11-15

**Authors:** Zofia Strojny, Rafał Rutkowski, Alina Kanikowska, Agnieszka Zawada, Aldona Juchacz, Marian Grzymisławski, Maki Sato, Monika Litwinowicz, Katarzyna Korybalska, Andrzej Bręborowicz, Janusz Witowski, Dominika Kanikowska

**Affiliations:** 1 Department of Pathophysiology, Poznan University of Medical Sciences, 61-701 Poznan, Poland; zstrojny@ump.edu.pl (Z.S.); rrutkowski@ump.edu.pl (R.R.); mlitwinowicz@ump.edu.pl (M.L.); koryb@ump.edu.pl (K.K.); abreb@ump.edu.pl (A.B.); jwitow@ump.edu.pl (J.W.); 2 Department of Gastroenterology, Dietetics and Internal Diseases, Poznan University of Medical Sciences, 60-355 Poznan, Poland; akanikowska@ump.edu.pl (A.K.); a.zawada@ump.edu.pl (A.Z.); ajuchacz@wcpit.org (A.J.); mariangrzym@ump.edu.pl (M.G.); 3 Department of Physiology, Institutional Research, School of Medicine, Aichi Medical University, Aichi 480-1195, Japan; msato@aichi-med-u.ac.jp

**Keywords:** chronotype, calorie restriction, obesity

## Abstract

Background: Chronotype is the pattern of the circadian rhythm that allows an individual to optimize times of sleep and activity. It has been observed that chronotypes may associate with some conditions and diseases, including obesity. It is not known, however, whether chronotypes determine the effectiveness of weight loss regimens. Therefore, in the present study, we compared the outcomes of a 3-week moderate calorie restriction undertaken by individuals with obesity under the same controlled hospital conditions. Methods: A total of 131 participants with obesity (median BMI 40.0) were studied. The subjects underwent the same dietary intervention over 3 weeks, with a 30% reduction in daily caloric intake. The individual chronotypes were assessed by the morning and evening questionnaire (MEQ) according to Horne and Östberg. Anthropometric and biochemical parameters were assessed by routine methods. Results: Of all patients examined, 75% had the morning (lark) chronotype and 25% had the evening (owl) chronotype. These patient sub-groups did not differ in terms of demographic, anthropometric and biochemical characteristics at baseline. After 3 weeks of calorie restriction, both groups experienced a similar loss of weight and BMI (Body Mass Index) (3.4 ± 0.38% for larks vs. 4.1 ± 0.47% for owls, *p* = 0.45), with owls exhibiting a marginally greater loss of body fat (3.1 ± 0.79%) compared with larks (2.6 ± 0.64%), *p* = 0.02. On the other hand, the larks had a more discernable, but not statistically significant from owls, decrease in glycated haemoglobin and CRP (C Reactive Protein). Conclusions: The chronotype of individuals with obesity does not have a significant effect on the magnitude of the body weight loss, but there is a tendency observed towards the reduction in body fat content in owls through changing their meal and sleep timing to earlier hours, in response to moderate calorie restriction applied under the same controlled conditions.

## 1. Introduction

The chronotype is a pattern of the circadian rhythm that determines the most optimal activity and rest time. It is divided into morning (“lark”), evening (“owl”), and mixed type.

It seems indisputable that there is a close correlation between the chronotype and health condition, including the incidence of specific diseases [1,2,3].

Based on the relationship between chronotype and obesity, people defined as evening type present a predisposition to an increased adipose tissue content [4]. Numerous studies suggest that this relationship may be determined by diet. Maukonen (2016) noted that people with the evening chronotype were much more likely to lead an unhealthy lifestyle while not showing a greater genetic predisposition to obesity compared to individuals from other chronotypes [5]. Similar conclusions were also reached by Lucassen et al., who determined that evening chronotype individuals were associated with the later consumption of meals, more meals per day, night snacking, and a higher level of stress hormone and the risk of cardiovascular events related to obesity [6].

Considering the scientific data, few reports exist on the relationship between chronotype and the effectiveness of a diet. Ross et al. focused their studies on the effectiveness of a reduction diet, taking into account the subjects’ chronotype. Of the subjects studied, the majority (73%) were women. During the course of the study, the authors tried to determine whether the chronotype of the subject affected the results of the diet used. It was found that the “morning” chronotype individuals had a higher chance of weight loss. This phenomenon was determined by longer sleep and its better quality [7]. In the Ross experiment, participants were not subject to the same dietary restrictions but were recruited as members of the National Weight Control Registry (NWCR) who lost at least 13.6 kg and maintained that weight for at least 1 year.

Furthermore, we investigated a soluble form of the advanced glycation end products receptor (sRAGE) [8]. RAGE receptors show a strong relationship with obesity [9], and their expression within adipocytes is higher in the population with increased body weight [10]. The available literature on the subject provides ample evidence indicating a close relationship between the concentration of the RAGE receptor’s soluble form and body weight [11,12]. Moreover, many authors suggest that sRAGE may be considered a universal biomarker used to treat obesity and its complications [9,13].

Although prior studies have focused on the eating and physical activity patterns of successful weight loss, there has been no research to date on the chronotype in control condition. As many individuals may have difficulties in adhering to prolonged and substantial caloric restriction resulting in a significant weight loss, here we have addressed this issue in a setting more likely to reflect a real-life situation but in hospital conditions, and assessed various biochemical parameters in obese volunteers undergoing only short-term (3 weeks) and rather moderate calorie restriction (25–30% energy deficit).

The aim of the study was to determine whether the individual chronotype is related to the weight loss.

## 2. Materials and Methods

The study protocol was approved by the Bioethics Committee of the Medical University of Poznań (Resolution No. 201/19) and was in line with the Helsinki Declaration’s basic principles. Each subject signed a consent form to participate in the study and was informed in detail about its course.

### 2.1. Study Group

The study group consisted of 131 people (61 men and 70 women) treated for obesity at the Department and Clinic of Internal Diseases, Metabolic Diseases, and Dietetics of the Medical University of Poznań, outlined in Table 1. The subjects were individuals with obesity, falling into pre-defined criteria with BMI (Body Mass Index) >30 kg/m^2^, age >18 years old, who were invited to initiate a weight loss regimen on a doctor’s advice. The exclusion criteria for the study included: vegetarian (or another alternative) diet consumption; previous or current neoplastic disease (receiving radiotherapy, chemotherapy); cardiovascular, autoimmune, congenital metabolic or liver diseases; inflammatory bowel disease (Crohn’s disease, ulcerative colitis); status after ischemic or hemorrhagic stroke (<6 months) and after STEMI (ST Elevation Myocardial Infarction) or NSTEMI (No ST Elevation Myocardial Infarction) (<12 months); eating disorders (anorexia, bulimia); mental disorders; alcohol/drug abuse; and ongoing antibiotic therapy and steroid therapy.

Individuals who qualified for the study were subjected to a three-week dietary treatment in a hospital setting. All patients were hospitalized and subjected to the same environmental factors, schedule of the day, amount of physical exercise, and catering diet including vegetables, meat, dairy, and grain products. They used an individually selected diet with a 25–30% reduction in the daily caloric supply (reduction of 500 to 1000 kcal) in relation to the total energy requirement, calculated according to the Harris and Benedict formula [14] and the physical activity index. All patients received the same type of diet provided by a catering company, with the same proportion of nutrients: 20% protein, 25–30% fat, and 50–55% carbohydrates.

The patients’ daily schedule was as follows:

- 7.00 am—wake up;
- 7.30 am—breakfast;
- 8.30 am—breathing exercises 30 min;
- 9.30 am—strengthening exercises 30 min;
- 10.15 am—brunch;
- 11.00 am—stationary bike ride 40 min;
- 12.00 pm—meeting with a dietitian;
- 12.40 pm—lunch;
- 13.30 pm—breathing exercises 30 min;
- 17.00 pm—dinner;
- 23:00 pm—bedtime.

During their stay at the Department and Clinic of Internal Diseases, Metabolic Diseases and Dietetics of the Medical University of Poznań, the patients had two blood samples taken for biochemical tests, based on which the levels of glucose, insulin, C-reactive protein, HbA1c glycated hemoglobin in the blood serum, and insulin resistance were calculated. Sampling was performed at the same time of day (between 07:00 h and 08:00 h) and in a fasting state to avoid circadian influences.

Samples were centrifuged and stored at −80 °C until assay. Each sample was assayed for soluble receptor for advanced glycation end products (sRAGE) using enzyme-linked immunosorbent assays (ELISA) using the manufacturers’ instructions (R&D system, Minneapolis, USA). The sensitivity of the sRAGE was 48 pg/mL. The routine biochemical investigations were carried out in the University Hospital’s central laboratory, using standard commercial reagent kits. Bodyweight measurement was also conducted, including body composition analysis using the BIA electrical bioimpedance method, which allowed the estimation of both the percentage and weight of adipose tissue and muscle tissue in patients. The measurement was performed under standardized conditions (in the morning, in the fasting state, with an empty bladder, and with the body in a standardized spatial position).

Simultaneously, all participants completed the morning and evening questionnaire (MEQ) according to Horne and Östberg (1976) to assess individual daily preferences [15]. On this basis, they were divided according to the presented type of chronotype: lark (scoring ≥ 59), owl (scoring ≤ 41), and intermediate type (scoring 42–58).

However, ultimately it was decided to combine the last two types; therefore, owl and intermediate were classified as the evening type [4,16].

### 2.2. Statistical Analysis

The calculations were made using the Statsoft Statistica 12 program. The level of significance was set at α = 0.05. The result was considered statistically significant when *p* < α. The normality of the distribution of variables was checked using the Shapiro–Wilk test. The Mann–Whitney test was used to calculate the two groups of variables. To test the relationship between continuous variables, Spearman’s rank correlation coefficient was calculated, and in the case of categorical variables, the chi2 test of independence. In order to analyze changes over time, for related samples, the Wilcoxon test was used. The sRAGE results were assessed in terms of outliers’ values in the Grubbs’ test. After removing two outliers above 2000 pg/mL, the statistical significance did not change significantly. Therefore, in the entire further study, the sRAGE results were presented with outliers included. The values of the results in the study are given as a median (minimum–maximum).

## 3. Results

**Figure 1:**
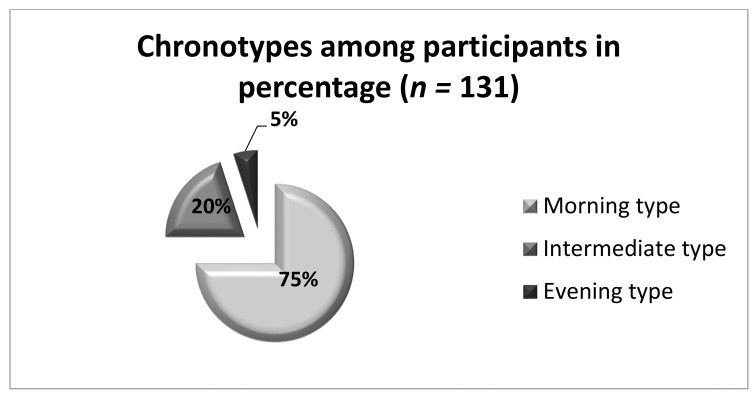
Distribution of chronotypes among all participants, *n* = 131.

Of 131 patients examined, 98 (75%) were found to have the morning chronotype, 7 (5%) had the evening chronotype, and 26 (20%) had the intermediate chronotype (Figure 1). Due to the small number of subjects with the pure evening chronotype, for the purpose of this analysis the participants with intermediate and evening chronotypes were grouped together and designated further as owls. These patients were compared to those with the morning chronotype, designated as larks. Such an approach was used previously in other studies [16,17].

The participants’ characteristics are given in Table 1. At the beginning of the study, there were no differences between larks and owls in terms of basic demographic and anthropometric criteria. There were also no significant differences between the groups in biochemical parameters related to glycemic control and inflammation (blood glucose and insulin, insulin resistance, glycated haemoglobin, and soluble receptor for advanced glycation end-products) (Table 2).

**Figure 2:**
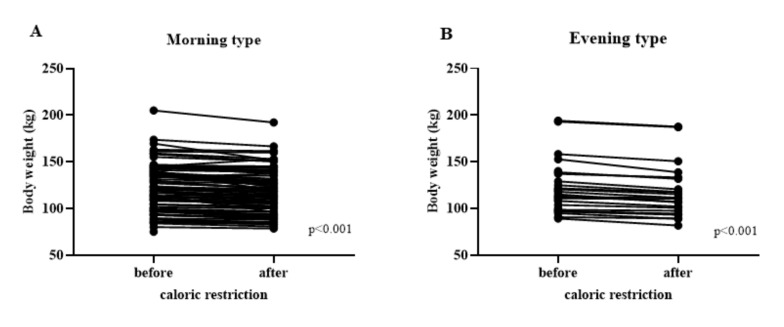
Body weight before and after caloric restriction. Wilcoxon signed-rank test. Individual datapoints linked by a line for: (**A**)—morning chronotype (*n* = 98), (**B**)—evening chronotype (*n* = 33).

**Figure 3:**
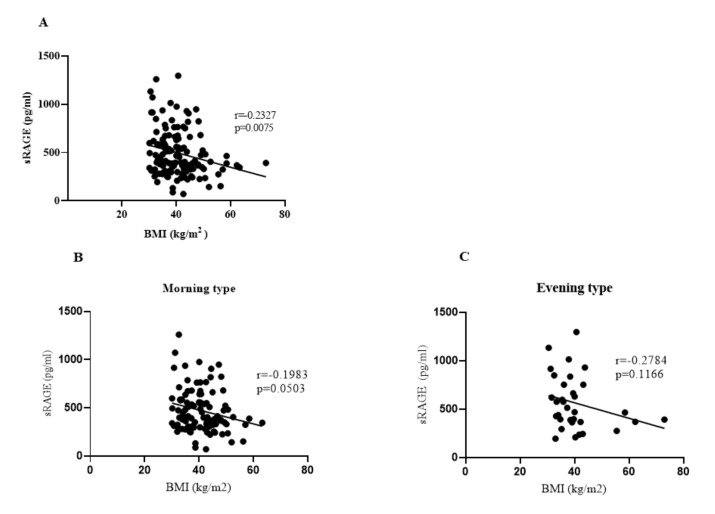
Correlation between serum sRAGE concentration (pg/mL) and BMI (kg/m^2^) before caloric restriction; Spearman test. (**A**) In the study group, *n* = 131. (**B**,**C**) In each chronotype: (**B**)—morning type (*n* = 98), (**C**)—evening type (*n* = 33).

The 3-week calorie restriction resulted in a a modest, but significant reduction in body weight (Figure 2), and consistently in BMI, % of adipose tissue, and visceral fat. While there was no difference between larks and owls in the magnitude of the reduction in weight or BMI, there was a marginally greater loss of body fat in owls (3.1 ± 0.79%) compared with larks (2.6 ± 0.64%), *p* = 0.02 (Table 2). In addition, the larks had a clearer decrease in glycated haemoglobin and CRP (C Reactive Protein), but this did not differ significantly from that in owls (Table 2). Since it has previously been suggested that the concentration of sRAGE decreases in obesity [7,18], we assessed sRAGE levels in our patients. Indeed, we observed a weak, inverse correspondence between BMI and sRAGE concentration (r = −0.2327, *p* = 0.0075; Figure 3A), which, however, did not differ between larks and owls (Figure 3B,C). Moreover, a decrease in BMI following the dietary intervention was not associated with a significant change in sRAGE concentration in either group.

## 4. Discussion

To the best of our knowledge, this is the first study to assess the relationship between dietary restriction and chronotype. Due to its innovation and pioneering nature, this aspect seems particularly interesting to us in exploring obesity. Imposed dietary schedules allow us to observe if, according to chronotype, the same timing and caloric load of meals results in different weight reduction. This type of restricted conditions removes differences due to chronotype eating habits, which, according to the literature, appears to be less healthy in the owl chronotype, consuming more food in the evening.

Research provides ample evidence that the chronotype is associated with the occurrence of specific metabolic disorders [1,3]. Underlying these aberrations are disturbances in biological rhythms that control sleep and wakefulness [19].

When analyzing the study group, we found that 75% of obese patients were larks, while 25% were owls, a correlation that is opposite to that reported in the literature [20,21]. The prevalence of participants with a lark chronotype may suggest that this type of chronotype is more eager to take care of their health and search for medical help. According to Dashti et al., morning diurnal preference is connected with an increased intake of healthy foods and better adherence to recommendations [22].

Multiple authors note that people described as evening type present a predisposition to increased body fat content [4,23]. This dependence may be determined by the individuals’ diet and unhealthy lifestyle [5,6].

The dietary restriction we carried out in our study allowed for a significant reduction in body weight, BMI, % of adipose tissue, and visceral fat both in the case of the lark type and representatives of the owl type. Comparing these two studied groups, owls reduced body fat more significantly than larks, possibly because of imposed earlier meal consuming—last meal at 5 p.m. and regular bedtime an hour before midnight. It suggests that, indeed, late eating and falling asleep are connected with negative metabolic shifts, predisposing patients to greater body fat accumulation. Circadian misalignment seems to be harmful only if it takes place during night hours, as it is observed in many studies of night-shift workers [18,24]. The shift to earlier diurnal hours in our study had a beneficial effect on the faster reduction in body fat content in participants with late hours activity before the study period.

It should be noted that the nutritional modification carried out was effective in relation to other parameters: a decrease in CRP, HOMA-IR (Homeostasis Model Assessment of Insulin Resistance), or HbA1c (Glycated Haemoglobin). The improvement in insulin resistance associated with HOMA-IR reduction was recorded in the evening type (*p* = 0.0107), and the glycosylated hemoglobin and CRP decreased in the morning chronotype (*p* = 0.0047 and *p* = 0.0039, respectively).

This result is similar to the Meydani study, which also reported a 40% reduction in CRP following a long-term moderate diet [25].

In turn, Reutrakul et al., in their work, emphasized the potential role of the evening chronotype as a prognostic factor for disturbances in glycemic control and promoting the subsequent development of insulin resistance [3].

Additionally, it is worth emphasizing that multiple studies reported a negative correlation between body weight, BMI, and serum sRAGE concentration [9,12,13]. Considering this dependence, the effect of diet therapy may be an appealing new topic for researchers.

In our study, weight loss did not correlate with the change in sRAGE; sRAGE concentration did not change among all subjects (*p* = 0.7760) and after taking into account the division by chronotype. The limitation of the study is its short duration but, on the other hand, all participants were in the same supervised environmental circumstances, which enabled the assessment of the influence of extrinsic factors on different types of chronotype. These small sample sizes and the short-term caloric restriction regimen were imposed by the hospital conditions and require further confirmation in a larger cohort and for a more extended period followed by a weight-stable period.

More research is needed to define the chronotype’s exact role in the effectiveness of a diet.

## 5. Conclusions

Our research found that an individual’s chronotype is not related to body weight loss or concentration of the soluble form of the receptor for advanced glycation products (sRAGE), but there is a tendency observed towards a faster reduction in body fat content in owls by changing their meal and sleep timing to earlier hours compared to larks. All other parameters such as caloric load, food composition, and physical exercise/activity levels were the same. The results of the study suggest that taking into consideration the chronotype of obese patients may possibly facilitate a particular body fat content reduction by changing the timing of the meals, but further research is needed to assess other correlations between chronotype and factors influencing weight changes.

## Figures and Tables

**Table 1:** Anthropometric characteristics of all study participants by chronotype (*n* = 131); Kruskal–Wallis test. The values of the results in the study are given as a median (minimum–maximum). BMI (Body Mass Index).

Parameter	Morning Type (*n* = 98)	Evening Type (*n* = 33)	*p*
Age, years	47.0 (22.0–69.0)	38.0 (21.0–63.0)	0.0737
Body weight, kg	115.8 (75.0–205.0)	113.0 (89.2–194.0)	0.8011
BMI, kg/m^2^	40.5 (30.1–63.3)	38.5 (30.5–73.0)	0.2233
Female, n(%)	55 (56)	15(45)	
Post-menopausal female, n (%)	26 (27)	7 (21)	
Diabetes mellitus patients, n (%)	16 (16)	6 (18)	

**Table 2:** Anthropometric and metabolic characteristics of the study group (*n* = 131), taking into account measurement changes before and after caloric restriction, by chronotype; Wilcoxon signed-rank test. The values of the results in the study are given as a median (minimum–maximum). BMI (Body Mass Index), HOMA-IR (Homeostasis Model Assessment of Insulin Resistance), HbA1c (Glycated Haemoglobin), CRP (C Reactive Protein).

The Obese (*n* = 131) before and after Caloric Restriction, Broken Down by Chronotype									
Parameter	Morning Type before (M1) *n* = 98	Evening Type before (E1) *n* = 33	Morning Type after (M2) *n* = 98	Evening Type after (E2) *n* = 33	M1 vs. M2 *p*	E1 vs. E2 *p*	Δ (%) Morning Type	Δ (%) Evening Type	Δ (%) Morning Type vs. Δ (%) Evening Type *p*
Body weight, kg	115.8 (75.0–205.0)	113.0 (89.2–194.0)	115.2 (78.5–192.3)	110.9 (81.8–187.6)	<0.001	<0.001	3.4	4.1	0.4486
BMI, kg/m^2^	40.5 (30.1–63.3)	38.5 (30.5–73.0)	39.9 (29.0–59.4)	38.4 (30.4–70.6)	<0.001	<0.001	3.25	4.0	0.3298
Fat tissue, %	41.7 (25.8–51.0)	43.6 (27.1–51.1)	40.9 (21.3–50.1)	41.8 (24.5–51.4)	<0.001	0.0019	2.6	3.1	0.0224
Visceral fat, kg	16.0 (5.0–43.0)	14.0 (10.0–45.0)	14.0 (4.0–39.0)	13.0 (10.0–44.0)	<0.001	0.0033	6.7	7.1	0.7605
sRAGE serum, pg/mL	371.8 (70.4–1259.0)	394.1 (194.3–1295.0)	389.7 (74.5–1011.0)	533.9 (82.0–1343.0)	0.8078	0.2652	0.85	5.7	0.6779
Glucose, mg/dl	101.0 (79.0–308.0)	103.0 (79.0–290.0)	101.0 (82.0–203.0)	105.0 (78.0–253.0)	0.3972	0.3203	0.0	2.1	0.7438
Insulin, μU/L	18.6 (4.7–79.6)	17.1 (7.6–45.3)	13.9 (1.3–78.0)	12.7 (7.3–43.8)	0.1249	0.2845	7.8	8.0	0.6749
HOMA-IR	4.5 (1.2–25.2)	4.3 (1.8–11.4)	3.0 (0.0–50.4)	2.5 (0.0–10.4)	0.0591	0.0107	5.9	9.7	0.4370
HbA1c, %	5.8 (4.4–9.80	5.7 (4.8–9.6)	5.4 (4.4–16.0)	5.5 (4.7–8.8)	0.0047	0.0712	4.3	5.7	0.8387
CRP, mg/L	5.0 (0.5–22.5)	3.7 (0.5–13.4)	3.1 (0.5–35.2)	3.9 (0.5–11.3)	0.0039	0.3507	30	24.3	0.9183

## Data Availability

The data supporting the conclusions of this article are included within the manuscript. The dataset is available from the corresponding author on request.
